# Benign Metastasizing Leiomyoma in the Lung Presenting in a Phyllodes-Like Pattern Mimicking a Biphasic Tumor: A Case Report

**DOI:** 10.1177/10668969211035059

**Published:** 2021-07-21

**Authors:** Saleh Fadel, Patrick J. Villeneuve, Ashish Gupta, Sarah Strickland, Marcio Gomes

**Affiliations:** 1 The Ottawa Hospital, 12365 University of Ottawa, Ottawa, ON, Canada

**Keywords:** benign metastasizing leiomyoma, biphasic tumors of the lung, mimics of lung tumors, pulmonary tumors

## Abstract

Primary biphasic tumors of the lung are rare. Lung lesions with a biphasic pattern are far more commonly primary or metastatic soft tissue tumors with entrapped native respiratory epithelium, giving the false impression of a biphasic tumor. We report a case of bilateral benign metastasizing leiomyomas in a 69-year-old female where the tumor cells diffusely entrapped native respiratory glands in a phyllodes-like pattern. The radiographic characteristics and histologic appearance were not immediately diagnostic and covered a wide differential. Reaching the final diagnosis required the use of immunohistochemical studies as well as correlation with the patient's history and radiographic findings. To the best of our knowledge, this is the first report of pulmonary benign metastasizing leiomyoma presenting in a phyllodes-like pattern. This case illustrates the importance of considering entrapment of native lung epithelium in the differential diagnosis of biphasic-appearing lung tumors.

## Introduction

Primary biphasic tumors of the lung such as adenofibroma are exceptionally rare.^1^ Pulmonary neoplasms with a biphasic pattern more commonly correspond to a monophasic neoplasm, primary or metastatic, with entrapment of native respiratory epithelium. This phenomenon has only been well characterized recently in 2020 by Erber et al.^2^ The authors studied 23 such cases and showed that many types of primary and metastatic tumors can entrap native respiratory epithelium and appear like biphasic tumors.^2^ Their series included a wide variety of primary and metastatic tumors, including solitary fibrous tumors, germ cell tumors, and various sarcomas. Herein, we present a case of benign metastasizing leiomyoma to the lung with diffuse entrapment of native respiratory epithelium in a phyllodes-like pattern mimicking a biphasic tumor.

## Case Presentation

**Figure 1.**
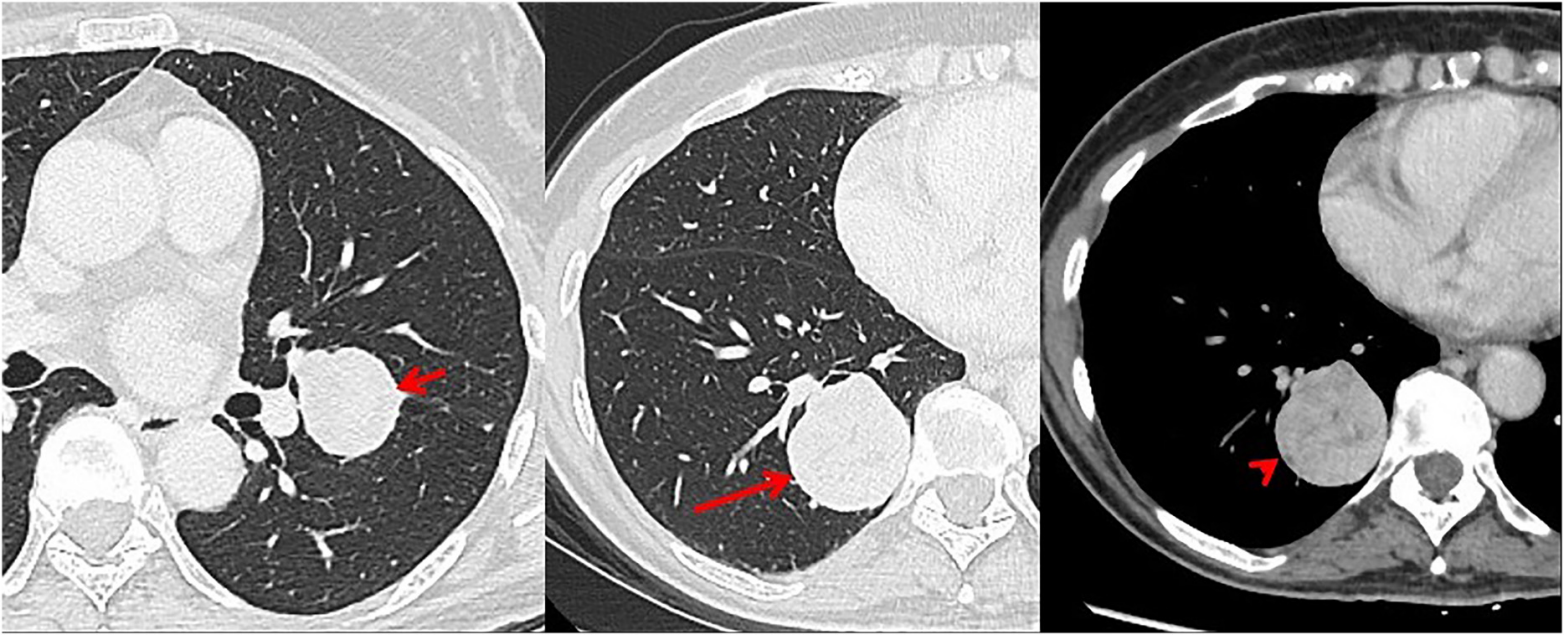
Computed tomography (CT) images in axial plane in lung window (A and B) show well defined, smooth outline solid left lower lobe nodule (short arrow) and right lower lobe lung mass (long arrow). On mediastinal windows (C), the right lower lung mass (arrowhead) shows slight heterogeneity and enhancement.

A 69-year-old female, never smoker, with a history of hysterectomy for benign fibroids performed 20 years earlier presented with back pain. A chest radiograph was performed as part of workup which revealed 2 lung lesions. A follow-up chest computed tomography (CT) with intravenous contrast confirmed the presence of a well-demarcated right lower lobe mass (4.2 × 3.8 cm) and a left lower lobe nodule (2.8 ×  2.6 cm) extending to the left upper lobe (Figure 1). The lung lesions were hypervascular and caused mild extrinsic compression over the adjacent bronchovascular bundle. Based on imaging, a wide differential diagnosis of primary lung tumors, including sclerosing hemangioma and carcinoid tumor, or metastatic deposits from hypervascular extrathoracic cancers, such as melanoma or renal cell carcinoma were considered. The patient was completely asymptomatic from a respiratory standpoint. Her pulmonary function tests revealed a forced expiratory volume in 1 second (FEV1) at 106% predicted and a diffusion capacity for carbon monoxide (DLCO) at 103% predicted. During workup, an abdominal CT showed a 1.4 ×  1.9 cm slowly growing hypervascular mass in the left adnexa. A transvaginal ultrasound demonstrated that it was separate from the left ovary. Of note, the patient was receiving estrogen replacement therapy.

The patient was referred to thoracic surgery and underwent an image-guided biopsy, which revealed a cellular spindle neoplasm with immunohistochemical findings supportive of a benign smooth muscle neoplasm. She then underwent a right lower lung lobectomy with lymph node dissection followed by a left lower lung lobectomy with lymph node dissection 2 months later.

**Figure 2.**
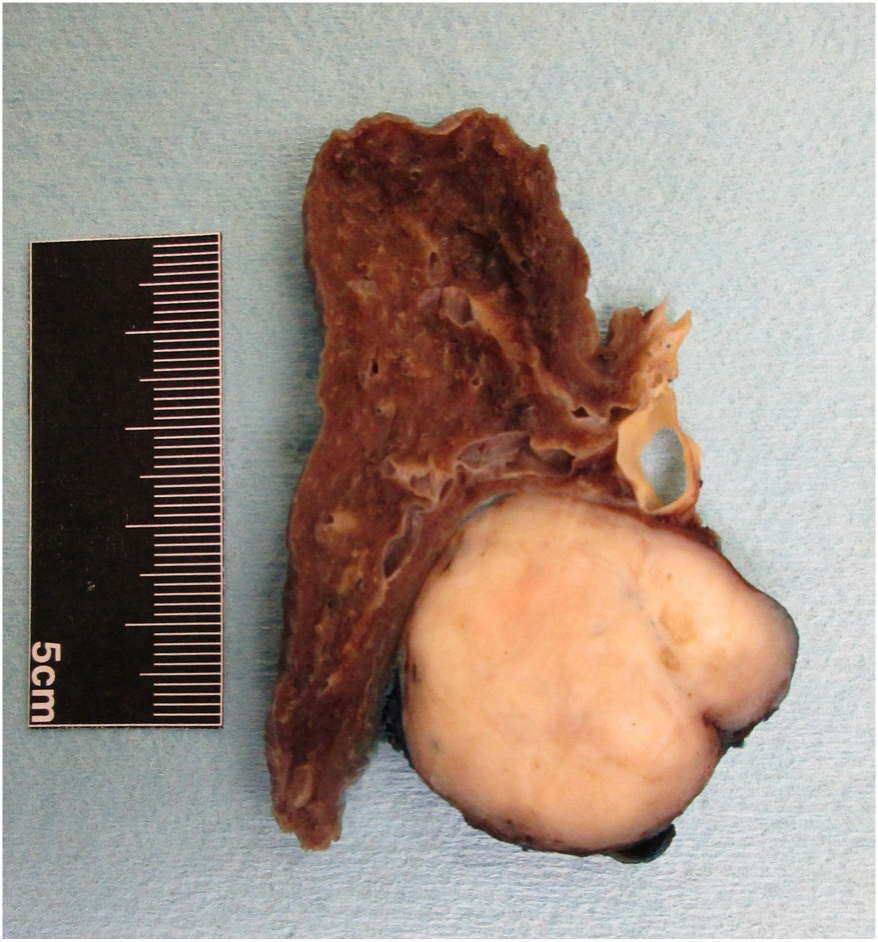
Gross image of the left lower lobe lesion, showing a round well-circumscribed white nodule compressing adjacent native structures. This was identical to the previously resected lesion from the right lower lobe.

**Figure 3.**
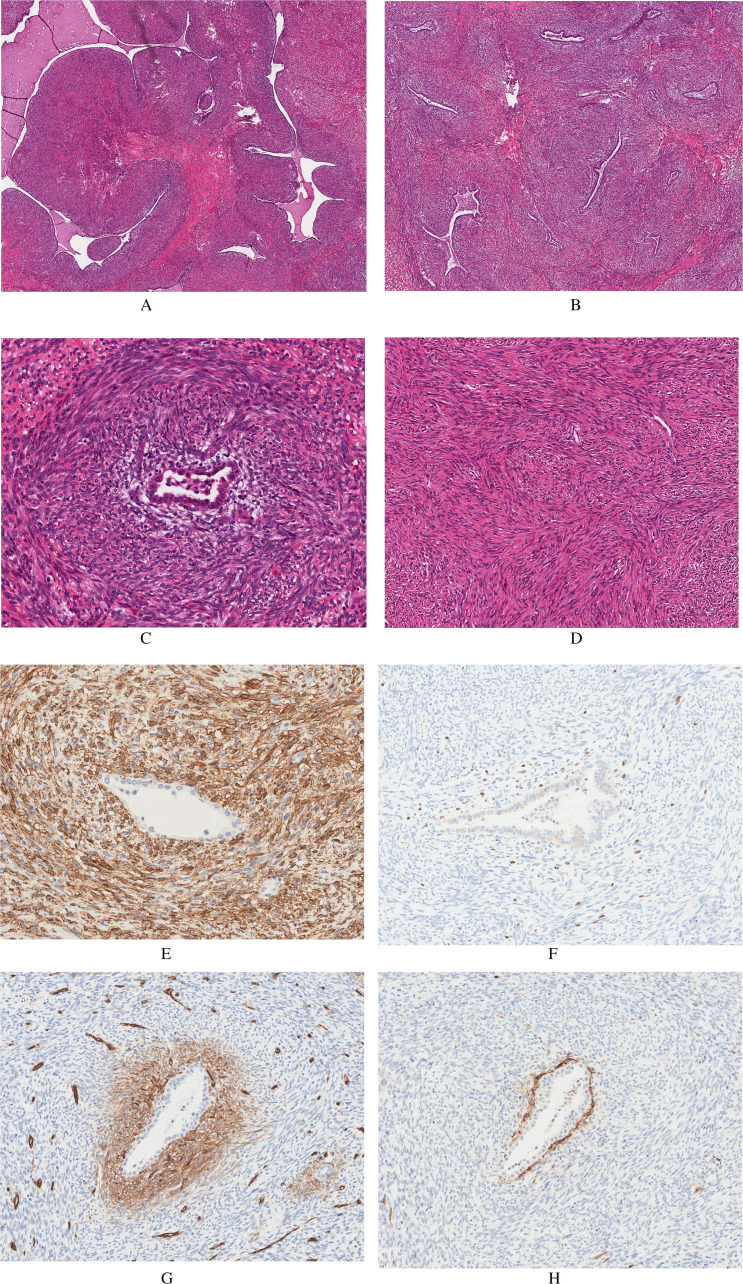
Representative images of the lesion from one histological section showing diffuse lung epithelium entrapment (A-D) and the results of select immunohistochemical studies (E-H). (A) Phyllodes-like pattern, (B) adenofibroma-like pattern, (C) a high power image of an entrapped respiratory gland with hypercellular stroma containing short spindle cells surrounding the epithelial gland, (D) the classic leiomyoma-like pattern, (E) smooth muscle actin expression in the spindle cell component, (F) elevated Ki-67 expression in the periglandular spindle cell area, (G) CD34 expression in the periglandular spindle cells, and (H) CD10 expression in the periglandular spindle cells.

The gross appearance of both lesions was identical, showing rounded and well-demarcated contours with compression of native nearby structures without invasion (Figure 2). Histologic examination of the right lower lobe lesion revealed a well-circumscribed neoplasm with a diffuse biphasic pattern. The tumor was composed of a spindle cell population with leaf-like projections lined by glandular epithelium in a phyllodes-like pattern (Figure 3A). Some areas showed spindle cells with patchy islands of glandular epithelium arranged in an adenofibroma-like pattern (Figure 3B). 3 types of spindle cells were noted: predominant short bipolar spindle cells with cigar-shaped nuclei and scanty cytoplasm; long bipolar spindle cells with cigar-shaped nuclei and eosinophilic cytoplasm of classic smooth muscle morphology; and focal epithelioid spindle cells with abundant eosinophilic cytoplasm. There was no significant nuclear atypia or pleomorphism, no areas of necrosis, and the mitotic count was low (2 mitoses per 10 mm^2^ or 0.4 per 2 mm^2^). The short spindle cells concentrated around the epithelial glandular cells and formed hypercellular areas with short interwoven fascicles (Figure 3C). The epithelial cells were hobnail to cuboidal and showed reactive changes, but no dysplasia. One of the histological sections showed a focal area of classic benign leiomyoma with pure spindle cell morphology (Figure 3D). The morphological differential diagnosis included biphasic pulmonary neoplasms (eg, adenofibroma and pulmonary leiomyomatous hamartoma), metastatic biphasic neoplasms (eg, phyllodes tumor of the breast and adenosarcoma of gynecological tract), and primary or metastatic spindle cell neoplasms with entrapped respiratory epithelium.

Immunohistochemical studies showed that the spindle cell component was positive for desmin, smooth muscle actin (SMA) (Figure 3E), H-caldesmon, estrogen, and progesterone receptors (diffuse, strong), cluster of differentiation 99 (CD99) and B-cell lymphoma 2 (BCL-2). The overall Ki-67 labeling index was ∼2%, but up to 10% in the hypercellular spindle cell component surrounding the epithelial islands (Figure 3F). Interestingly, this periglandular spindle cell component was positive for CD34 and CD10 (Figure 3G and H, respectively). The focal area with classic leiomyoma morphology showed a similar immunophenotype with some variation in the intensity of staining. The epithelial component was positive for pan-cytokeratin, epithelial membrane antigen (EMA), and thyroid transcription factor 1 (TTF-1). Both components were negative for S100 protein, cluster of differentiation 117 (CD117), signal transducer and activator of transcription 6 (STAT-6), paired-box gene 8 (PAX-8), gross cystic disease fluid protein 15 (GCDFP-15), and GATA binding protein 3 (GATA-3).

The morphological features and immunophenotype of the spindle cell component were consistent with a benign leiomyoma and the diffuse expression of estrogen and progesterone receptors suggested a uterine origin. The features of the epithelial component were consistent with nonneoplastic entrapped respiratory epithelium. The patient's previous hysterectomy specimen was reviewed. The uterus was removed intact without morcellation and was confirmed to show leiomyomas with no evidence of leiomyosarcoma or features of smooth muscle tumor of uncertain malignant potential. The immunohistochemical features were also similar to the stromal component of the lung lesion, including expression of SMA, desmin, H-caldesmon, estrogen, and progesterone receptors, CD99 and BCL-2. Markers of inflammatory myofibroblastic tumor (anaplastic lymphoma kinase (ALK) protein) and perivascular epithelioid tumor (human melanoma black (HMB) 45 and Mel-A) were negative. These findings, along with the presence of bilateral lung lesions with similar radiologic characteristics, were supportive of a benign metastasizing leiomyoma.

Histologic examination of the left lower lobe lesion showed similar features, but the entrapped lung epithelium was limited to a few adenofibroma-like glands which were mainly concentrated in the periphery.

Postoperatively the patient recovered well and had no radiological evidence of recurrence in the lungs at 7 months follow-up. Unfortunately, the patient developed urinary urgency and left-sided pelvic pain. A pelvic magnetic resonance imaging demonstrated growth of the previously noted left pelvic mass to a size of 4.2 ×  3.2 cm. A biopsy of this mass showed features consistent with benign metastasizing leiomyoma. Given that these tumors have been shown to be responsive to hormone modulation,^3^ she was advised to stop hormone replacement therapy and is now being followed with imaging studies. The patient has provided consent for this manuscript.

## Discussion

Benign metastasizing leiomyomas (BMLs) are rare but well described in the literature. They most commonly occur in women of reproductive age and can metastasize to numerous distant organs including lung (most commonly), skin, bone, mediastinum, lymph nodes, skeletal muscle, heart, and retroperitoneum.^4^ BMLs usually occur many years after a hysterectomy, with a mean interval of 8.8 years as reported in a recent systematic review.^5^ These lesions are usually asymptomatic and incidentally found.^4-6^ Although our patient was older than usual BML patients, it is interesting to note that she had been taking estrogen replacement therapy, which may explain the growth of these lesions since BML has been known to be responsive to hormonal modulation.^3^

Entrapment of native epithelium by tumor cells can be particularly puzzling in the lung where native epithelium can display prominent reactive changes closely mimicking neoplastic elements. This can give the impression of a biphasic tumor, a potential diagnostic pitfall. Although lung entrapment has been reported in cases of pulmonary BML and is present on published images,^7-10^ no special attention has been given to this phenomenon except for a paper in 2005 by Yamazaki.^11^ In this detailed study, the author describes 3 pulmonary BML cases with an adenomyoma-like pattern, which is similar to the pattern we observed in the left lower lobe lesion. What is peculiar in our case is the diffuse phyllodes-like pattern of entrapment we observed in the right lower lobe lesion, which has not been mentioned in previous reports.^7-11^ It is interesting that 2 distinct patterns of epithelial entrapment were present in 2 separate lesions in the same patient. Another notable aspect of our case was the distinct periglandular morphology with elevated Ki-67 and expression of CD34 and CD10. This is consistent with findings from Yamazaki's study, where the author postulated that there might be inductive interactions of periglandular mesenchymal stromal cells causing metaplasia in the entrapped respiratory epithelial cells.^11^

It was not until recently that patterns of tumor entrapment of lung epithelium were well investigated. In 2020, Erber et al^2^ found that 23 out of 47 nonepithelial neoplasms in the lung showed entrapment of respiratory epithelium. Based on a detailed study of those 23 cases, they describe 4 patterns of lung entrapment: adenofibroma-like, adenomyoepithelioma-like, biphasic synovial sarcoma-like, and pulmonary blastoma-like. The adenofibroma-like pattern was described as “leaflet-like variably dilated glands throughout the lesion imparting a characteristic adenofibroma-like, phyllodes-like or fibroepithelial hamartoma-like pattern” with diffuse entrapment of lung tissue,^2^ similar to our case. Only 1 out of their 11 cases with this pattern qualified as a true primary lung adenofibroma. The remaining 10 cases included a variety of primary and metastatic lesions, but none of them was a BML. As in our case, they noted that the entrapped glands frequently showed reactive/regenerative appearance with occasional hobnail-like nuclear changes, which can mimic neoplastic glands, but they lacked significant cytological atypia. To the best of our knowledge, this is the first description of a BML presenting with diffuse entrapment of lung epithelium in a phyllodes-like biphasic pattern.

## Conclusion

We present a case of bilateral BMLs in a 69-year-old female in which one of the tumors diffusely entrapped native respiratory epithelium in a phyllodes-like fashion mimicking a biphasic tumor. In accordance with previous studies, our case demonstrates the importance of considering a monophasic tumor, primary or metastatic, with entrapped native epithelium when encountering a lung tumor with biphasic architecture. It is also important to consider the patient's history and radiographic findings. If these are not available, it is appropriate to recommend further clinical investigations and imaging before diagnosing such a lesion as a primary biphasic lung tumor.
